# Location of Mental Foramen in a Selected Iranian Population: A CBCT Assessment 

**Published:** 2015-03-18

**Authors:** Leila Khojastepour, Sanam Mirbeigi, Sabah Mirhadi, Ateieh Safaee

**Affiliations:** a *Department of Oral and Maxillofacial Radiology, Dental School, Shiraz University of Medical Science, Shiraz, Iran;*; b * Department of Oral and Maxillofacial Radiology, Dental School, Shahid Sadoughi University of Medical Sciences, Yazd, Iran*

**Keywords:** Accessory Mental Foramen, Anatomic Landmarks, CBCT, Cone-Beam Computed Tomography, Mandible, Mental Foramen

## Abstract

**Introduction: **Mental foramen (MF) is an important anatomic landmark in dentistry and knowledge about its variable locations (L) and type of emergence (TE), has an effect on the sufficiency of local anesthesia and safety of surgical procedures. The aim of this study was to evaluate the L and TE of this radiographic landmark as well as the presence of accessory MF, by means of cone-beam computed tomography (CBCT). **Methods and Materials: **In this cross sectional study, a total of 156 CBCT images were retrieved from the archive of a private radiology clinic and were then evaluated for the position of MF and its TE and the existence of accessory foramina in the body of mandible. The extracted information was compared in both genders, in both sides of mandible and among three different age groups (20-29, 30-44 and 45-59 years). The Pearson chi-square and Fisher’s Exact tests were used for statistical analysis. The level of significance was set at 0.05. **Results: **Second premolar was the most common anterolateral L of MF; in general, 48.7% of right and 51.9% of left MFs were located at the apex of second premolar. Anterior and straight ET were more common in right and left side, respectively. Accessory MF was present in only 8 (5.1%) of cases. **Conclusion**: The possible presence of accessory MF should not be overlooked for avoiding the occurrence of a neurosensory disturbance during surgery and implant insertion.

## Introduction

Anatomy of the mandible and possible variations in position, course and type of emergence of its neurovascular bundle is important in gaining local anesthesia and during surgical procedures [1]. Implant placement in mandibular premolar region is one of the most complicated surgical procedures due to potential inadvertent complications of neurosensory alterations in the chin and lower lip that are likely to occur if mental foramen (MF) is not properly identified and protected [2]. The chance of damage to the neurovascular bundles exiting the MF is rather high after endodontic/orthogenetic surgery and fixation of bone fractures or surgical removal of roots, teeth, cyst and tumors [1]. MF is located in anterolateral aspect of mandible at an approximately equal distance (13-15 mm) from the superior and inferior border of the mandible [3]. Mental nerve and associated blood vessels pass through this foramen. The location (L) and emergence type (ET) of mental nerve has been described for a long time [4-6]. Recently this issue has again become the center of interest due to the need for accurate preoperative surgical planning for the placement of mandibular implants [1, 7-15]. It has been recommended to consider a 2-mm distance between the implant and the margin of the MF [6]. Ethnical differences between different nations play an important role in this regard [9, 15-17].

According to Pyun *et al.* [8], the L of MF could be classified into four groups based on its anteroposterior position: *Type 1* below the apex of second premolar; *Type 2 *between the apices of second and first premolars; *Type 3* between the apices of second premolar and first molar and *Type 4* distal to apex of first molar. 

**Figure 1:**
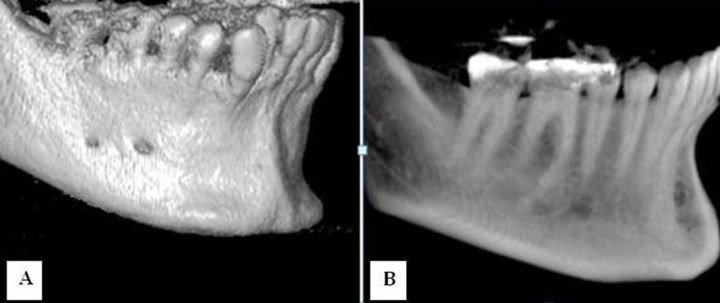
CBCT images show the mental foramen and accessory mental foramen below the apex of second premolar and first molar, respectively; *A*) volume style and *B*) ray cast style

Kieser *et al.* [15] classified the ET of mental nerve into four categories: posterior, anterior, right-angled or multiple-angled and found that a posterior direction was the most common ET. Recently Iyengar *et al. *[18], categorized the pattern of entry of mental nerve into the MF as straight, looping or perpendicular. 

Accessory MF (AMF) has different descriptions in articles. Some authors described AMF as any additional foramina except the main MF [3, 9]. On the other hand, in some articles only those foramina that are integrated with mandibular canal are nominated as AMF and the others are recognized as nutritional canals [19-22]. In some researches, any additional foramen except the MF has been named the buccal mandibular foramen [18, 21]. Oliveira *et al.* [23] reported accompaniment between AMF and bifid mandibular canal. The risk of hemorrhage, postoperative pain and paralysis could be reduced by detection of AMF prior to endodontic and surgical treatments including bone graft, implant insertion and osteotomy. Ethnic variations were reported to have a role in presence of AMF [3, 19, 20, 22, 24, 25].

The crucial benefit of cone-beam computed tomography (CBCT) is overcoming the limitations of conventional radiography by producing three-dimensional (3D) images that allow a comprehensive evaluation of the anatomy of the region of interest (ROI) [26]. The aim of this cross sectional study was to assess the L and ET of MF and presence of AMF in a selected Iranian population and to evaluate the effect(s) of patient’s age and gender on these variables, by means of CBCT.

## Materials and Methods

In this cross sectional study, 156 CBCT images that were acquired during a one-year period from archives of a private maxillofacial radiology clinic in Shiraz, Iran, were evaluated. The images were taken by NewTom VGi (QR SRL Co., Verona, Italy) with 110 kVp, 20 mA and voxel size of 0.3×0.3×0.3. The CBCT images were taken with different fields of view (FOV) (8×8 ,12×8 ,15×12 and 15×15) and were used only if they covered the ROI and matched the following inclusion criteria: Presence of first and second premolars in both sides of the mandible, absence of any pathology (radiolucencies that might represent cyst and tumor or periapical lesion), fracture, supernumerary or impacted teeth in ROI, images with high geometric resolution and availability of precise information about patient’s age and gender. 

CBCT scans were evaluated in terms of L, TE and presence of AMF. The L was recorded based on reconstructed 3D images (Figure 1). Considering the description of Kieser *et al.* [15], the ET of MF in the mandibular body were recorded based on CBCT axial cross-sections.

The frequency of the L in relation to age and gender was first analyzed descriptively. The Pearson chi-square and Fisher’s exact tests were used to assess the relation between patients’ demographic variables with L, ET and occurrence of AMF. Data were analyzed with SPSS software (SPSS version 15.0, SPSS, Chicago, IL, USA) and the level of significance was set at 0.05. 

**Table 1:** Location [N (%)] of Mental Foramen in different gender and age groups (P1: first premolar, P2: second premolar and M1: first molar)

**Variables**		**Right**			***P-*** **value**	**Left**			***P-*** **value**
		**P2**	**P1-P2**	**P2-M1**		**P2**	**P1–P2**	**P2-M1**	
**Gender**	**Male**	29 (42)	23 (33.3)	17 (24.6)	0.21	29 (42)	26 (37.7)	14 (20.3)	0.04^*^
	**Female**	47 (54)	27 (31)	13 (14.9)		52 (59.8)	27 (31)	8 (9.2)	
**Age (years)**	**20-29**	14 (43.8)	11 (34.4)	7 (21.95)	0.81	17(53.1)	12 (37.5)	3 (9.4)	0.60
	**30-44**	42 (51.2)	27 (32.9)	13 (15.9)		44 (53.7)	28 (34.1)	10 (12.2)	
	**45-59**	20 (47.6)	12 (28.6)	10 (23.8)		20 (47.6)	13 (31)	9 (21.4)	

**Table 2:** Type [N (%)] of mental nerve emergence to the mandibular body in different gender and age groups

**Variables**		**Right**			***P-value***	**Left**			***P-value***
		**Posterior**	**Anterior**	**Straight**		**Posterior**	**Anterior**	**Straight**	
**Gender**	**Male**	13 (18.8)	27 (39.1)	29 (42.0)	0.68	12 (17.4)	21 (30.4)	36 (52.2)	0.21
	**Female**	14 (16.1)	40 (46.0)	33 (37.9)		25 (28.7)	26 (29.9)	36 (41.4)	
**Age (years)**	**20-29**	2 (6.3)	12 (37.5)	18 (56.3)	0.19	5 (15.6)	9 (28.1)	18 (56.3)	0.42
	**30-44**	16 (19.5)	36 (43.9)	30 (36.6)		24 (29.3)	25 (30.5)	33 (40.2)	
	**45-59**	9 (21.4)	19 (45.2)	14 (33.3)		8 (19.0)	13 (31.0)	21 (50.0)	

**Figure 2:**
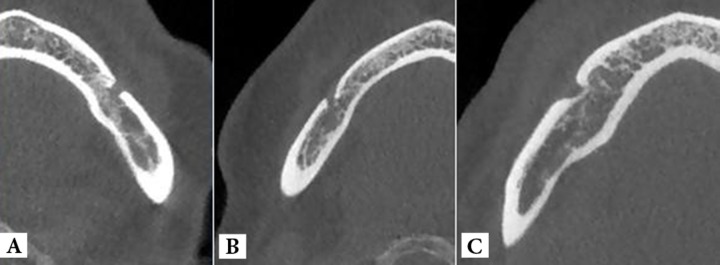
Mandibular canal emerging to mental foramen on surface of mandibular body in axial CBCT images. There are three emergence types; *A*) straight, *B*) anterior and *C*) posterior

## Results

Among 920 obtained CBCT images, a total of 764 scans were excluded mainly due to incomplete coverage of the ROI and missing one or more premolars. The final evaluated images comprised of 156 CBCTs belonging to 69 males and 87 females, with mean age of 36.99±8 years. MF was not absent in any of the cases and only 8 cases had AMF.

In general 48.7% of right and 51.9% of left MFs were located at the apex of second premolars (type 1). As shown in Table 1, type 1 location was the most common site for MF in both genders, both sides and all age groups. It is followed by type 2 and type 3. In none of the cases the MF was located anterior to the first premolar or at the apex of first molar. Patients’ age did not significantly affect the L. However, there was a significant difference between L on the left side of the mandible and gender (*P*<0.05) (Table 1).

The frequency of ET in different sex and age groups are reported in Table 2. There was no statistically significant difference between different ETs in different genders and age groups. In right side of the mandible, anterior opening of mental canal (42.95%), was the most common followed by straight and posterior direction (39.74% and 17.31%, respectively). In the left side straight ET was seen in 46.2% of cases followed by anterior and posterior ET (30.1% and 23.7%, respectively).

**Table 3:** Occurrence [N (%)] of accessory mental foramen (AMF) in different gender and age groups

**Variables**		**Absent**	**Present**	***P-value***
**Sex**	**Male**	63 (91.3)	6 (8.7)	0.14
	**Female**	85 (97.7)	2 (2.3)	
**Age**	**20-29**	30 (93.8)	2 (6.3)	0.94
	**30-44**	78 (95.1)	4 (4.9)	
	**45-59**	40 (95.2 )	2 (4.8)	

Table 3 shows the occurrences of AMF in different genders and age groups. AMF was present in only 8 cases (6 males and 2 females) out of 156. There was no statistically significant difference between frequency of AMF in different sex and age groups. AMF was bilateral in one out of 8 cases.

## Discussion

In this study the L and ET of MF and presence of AMF have been assessed in a selected Iranian population. In 156 evaluated CBCT images the apical area of the second premolar was the most common anterolateral location for MF while anterior and straight ET were more common in right and left side, respectively.

MF is one of the most important landmarks of mandible and it has gained interest from different aspects. Determination of its location, shape, size and distance from the other anatomic landmarks and adjacent roots has been subject of many studies [9, 19, 25, 27]. From clinical point of view, sufficient local anesthesia for dental treatments and safety of surgical procedures in this area are affected by the clinician’s knowledge about the position of MF. It also could play a role in interpreting the anatomical landmarks in forensics [19].

In the present study, the incidence of AMF was 5.1% (8.7% in males and 2.3% in females) which is in accordance with previous studies [3, 20, 28]. In this study MF was detected in both sides of mandible in all evaluated cases. However, in other studies, absence of MF was rarely reported. Freitas *et al.* [29] investigated the absence of the MF in 1435 dry human half mandibles (total of 2870 halves) and reported that the absence of MF in the right side (0.06%) to be twice as much the left side (0.03%). Recently, Hasan *et al.* [30] also reported a case of bilateral absence of MF during routine dissection tutorials on dry human mandibles. 

Regarding the position of MF our findings were in accordance with previous studies in which the most common location of MF was below the apex of second premolar (type 1) followed by a position in between the first and second premolars (type 2) and distal to second premolar (type 3) [5, 10, 11, 31-36]. On the other hand many other studies reported that the MF was most commonly located somewhere between first and second mandibular premolars (type 2) [14, 37-40]. Position of MF is affected by the ethnical characteristics. Recently Santini and Alayan [17] reported their anthropometric study on the position of the MF based on evaluation of 76 Chinese, 46 European and 33 Indian skulls. The modal position of the foramen in the Chinese samples was in line with the long axis of the second premolar, while among Europeans and Indians it was in between the first and second premolar. They concluded that population-based differences occur in the position of MF. 

The result of the study by Zamani *et al. *[36] regarding the position of MF in the same ethnic group is in accordance with the present study. They evaluated the position of MF in panoramic radiographs of 150 patients including 64 males and 87 females referring to the Department of Oral Radiology of Isfahan Faculty of Dentistry and reported the type 1 location as the most prevalent site. 

Our results however, are slightly different from the results reported by Haghanifar and Rokouei [14] who evaluated the position of MF in a selected Iranian population. In their study which was based on 400 panoramic radiographs the position between the first and second premolars (type 2) was the most common location of MF (47.2%). Type 2 location with close percentage (46%) was placed in second rank and it was followed by type 3 (5.1%) and type 4, respectively. 

The slight differences in the same population could be explained by using different imaging techniques. Panoramic radiography provides a flat image of a curved structure and is not as accurate as CBCT in horizontal localization of objects especially in premolar region.

In contrast to Kieser *et al.* [15] who stated that the posterior direction was the most common ET, the present study detected this emergence profile as the least common type. This could be explain by our method as we recorded the path of emergence based on axial cross sections rather than direct visualization of the skull or cadaveric dissections.

Formation of MF is incomplete until twelfth week of embryonic life when the mental nerve divides into several branches [41]. According to Toh *et al.* [42] if mental nerve’s branching occurs prior to formation of MF, AMF can be formed. Apart from different descriptions for AMF, being aware of its probable existence is important and if neglected, pain, paralysis, and even parasthesia after surgical procedures are likely [3, 9, 20, 24, 25, 27, 41]. The presence of AMFs have been evaluated with different methods including macroscopic investigations on dry skulls, plane radiography (including periapical and panoramic views), and computed tomography (CT or CBCT). According to Singh and Srivastav [9], evaluation of dry mandible and visual inspection during dissection is the most accurate method for diagnosing the presence of AMF. In clinical situations however, CBCT could provide sufficient information about the possible presence of AMF with reasonable amount of radiation. The incidence of AMF varies in different ethnic groups. The highest incidence is reported in Negros and Maori males [15]. According to Balcioglu and Kocaelli [3] the presence of AMF is a rather rare anatomical variation with prevalence ranging from 1.4 to 10%. Their report also revealed that non-Caucasians may have a higher incidence of AMF than Caucasians.

Gershenson *et al. *[28] in a study on 525 dry mandibles and dissections in 50 cadavers, reported that there were single and multiple MF in 94.67% and 5.33% of the cases, respectively. Among them 4.3% of the mandibles had double MFs, 0.7% had triple and one mandible had 4 MFs on one side.

## Conclusion

CBCT is an effective tool for three-dimensional assessment of MF. The possible presence of AMF should be borne in mind to avoid the occurrence of a neurosensory disturbance/hemorrhage following surgical procedures. It can be concluded that the most prevalent location for MF is below the apex of second premolar. 

## References

[1] von Arx T, Friedli M, Sendi P, Lozanoff S, Bornstein MM (2013). Location and dimensions of the mental foramen: a radiographic analysis by using cone-beam computed tomography. J Endod.

[2] Juodzbalys G, Wang H-L, Sabalys G (2010). Anatomy of mandibular vital structures Part II: mandibular incisive canal, mental foramen and associated neurovascular bundles in relation with dental implantology. J Oral Maxillofac Res.

[3] Balcioglu HA, Kocaelli H (2009). Accessory mental foramen. N Am J Med Sci.

[4] Tebo HG, Telford IR (1950). An analysis of the variations in position of the mental foramen. The Anatomical Record.

[5] Montagu M (1954). The direction and position of the mental foramen in the great apes and man. Am J Phys Anthropo.

[6] Riesenfeld A (1956). Multiple infraobaital, ethmoidal, and mental foramina in the races of man. Am J Phys Anthropol.

[7] Greenstein G, Tarnow D (2006). The mental foramen and nerve: clinical and anatomical factors related to dental implant placement: a literature review. J Periodontol.

[8] Pyun JH, Lim YJ, Kim MJ, Ahn SJ, Kim J (2013). Position of the mental foramen on panoramic radiographs and its relation to the horizontal course of the mandibular canal: a computed tomographic analysis. Clin Oral Implants Res.

[9] Singh R, Srivastav A (2010). Study of position, shape, size and incidence of mental foramen and accessory mental foramen in Indian adult human skulls. Int J Morphol.

[10] Chkoura A, El Wady W (2013). Position of the mental foramen in a Moroccan population: A radiographic study. Imaging Sci Dent.

[11] Udhaya K, Saraladevi K, Sridhar J (2013). The Morphometric Analysis of the Mental Foramen in Adult Dry Human Mandibles: A Study on the South Indian Population. J Clin Diagn Res: JCDR.

[12] Gupta S, Soni JS (2012). Study of anatomical variations and incidence of mental foramen and accessory mental foramen in dry human mandibles. NJMR.

[13] Smajilagić A, Dilberović F (2004). Clinical and anatomy study of the human mental foramen. Bosn J Basic Med Sci.

[14] Haghanifar S, Rokouei M (2009). Radiographic evaluation of the mental foramen in a selected Iranian population. Indian J Dent Re.

[15] Kieser J, Kuzmanovic D, Payne A, Dennison J, Herbison P (2002). Patterns of emergence of the human mental nerve. Arch Oral Biol.

[16] Cutright B, Quillopa N, Schubert W (2003). An anthropometric analysis of the key foramina for maxillofacial surgery. J Oral Maxillofac Surg.

[17] Santini A, Alayan I (2012). A comparative anthropometric study of the position of the mental foramen in three populations. Br Dent J.

[18] Iyengar AR, Patil S, Nagesh KS, Mehkri S, Manchanda A (2013). Detection of anterior loop and other patterns of entry of mental nerve into the mental foramen: A radiographic study in panoramic images. Journal of Dental Implants.

[19] Sisman Y, Sahman H, Sekerci A, Tokmak T, Aksu Y, Mavili E (2014). Detection and characterization of the mandibular accessory buccal foramen using CT.

[20] Naitoh M, Hiraiwa Y, Aimiya H, Gotoh K, Ariji E (2009). Accessory mental foramen assessment using cone-beam computed tomography. Oral Surg Oral Med Oral Pathol Oral Radiol Endod.

[21] Katakami K, Mishima A, Shiozaki K, Shimoda S, Hamada Y, Kobayashi K (2008). Characteristics of accessory mental foramina observed on limited cone-beam computed tomography images. J Endod.

[22] Naitoh M, Nakahara K, Hiraiwa Y, Aimiya H, GOTOH K, ARIJI E (2009). Observation of buccal foramen in mandibular body using cone-beam computed tomography. Okajimas Folia Anat Jpn.

[23] de Oliveira-Santos C, Souza PHC, de Azambuja Berti-Couto S, Stinkens L, Moyaert K, Rubira-Bullen IRF (2012). Assessment of variations of the mandibular canal through cone beam computed tomography. Clin Oral Investig.

[24] Mamatha N, Kedarnath N, Madhumathi Singh GP (2013). Accessory mental nerve: a case report. J Clin Diagn Res : JCDR.

[25] Parnia F, Moslehifard E, Hafezeqoran A, Mahboub F, Mojaver-Kahnamoui H (2012). Characteristics of anatomical landmarks in the mandibular interforaminal region: a cone-beam computed tomography study. Med Oral Patol Oral Cir Bucal.

[26] Kiarudi AH, Eghbal MJ, Safi Y, Aghdasi MM, Fazlyab M (2015). The Applications of Cone-Beam Computed Tomography in Endodontics: A Review of Literature. Iran Endod J.

[27] Leite GMF, Lana JP, de Carvalho Machado V, Manzi FR, Souza PEA, Horta MCR (2013). Anatomic variations and lesions of the mandibular canal detected by cone beam computed tomography. Surg Radiol Anat.

[28] Gershenson A, Nathan H, Luchansky E (1986). Mental foramen and mental nerve: changes with age. Cells Tissues Organs.

[29] Freitas V, Madeira M, Pinto C, Zorzetto N (1976). Direction of the mental canal in human mandibles. Aust Dent J.

[30] Hasan T, Fauzi M, Hasan D (2010). Bilateral absence of mental foramen—a rare variation. Int J Anat Variat.

[31] Ngeow WC, Yuzawati Y (2003). The location of the mental foramen in a selected Malay population. J Oral Sci.

[32] Mwaniki D, Hassanali J (1992). The position of mandibular and mental foramina in Kenyan African mandibles. East Afr Med J.

[33] Sankar DK, Bhanu SP, Susan P (2011). Morphometrical and morphological study of mental foramen in dry dentulous mandibles of South Andhra population of India. Indian J Dent Res.

[34] Miller J (1953). tudies on the location of the lingula, mandibular foramen, and mental foramen. J Dent Res.

[35] Philips jl, weller rn, kulild jc (1992). The mental foramen: Part II Radiographic position in relation to the mandibular second premolar. J Endod.

[36] Zamani Na, Hekmatian E, Rahmani L (2011). Frequency of horizontal position of mental foramina in the panoramic radiographs of patients referring to the Radiology Department of Isfahan Dental Faculty. Journal of Isfahan Dental School.

[37] Moiseiwitsch JR (1998). Position of the mental foramen in a North American, white population. Oral Surg Oral Med Oral Pathol Oral Radiol Endod.

[38] Al Jasser N, Nwoku A (1998). Radiographic study of the mental foramen in a selected Saudi population. Dentomaxillofac Radiol.

[39] Olasoji H, Tahir A, Ekanem A, Abubakar A (2004). Radiographic and anatomic locations of mental foramen in northern Nigerian adults. Niger Postgrad Med J.

[40] Fishel D, Buchner A, Hershkowith A, Kaffe I (1976). Roentgenologic study of the mental foramen. Oral Surg Oral Med Oral Pathol.

[41] Imada TSN, Fernandes LMPdS, Centurion BS, Oliveira‐Santos C, Honório HM, Rubira‐Bullen IRF (2014). Accessory mental foramina: prevalence, position and diameter assessed by cone‐beam computed tomography and digital panoramic radiographs. Clin Oral Implants Res.

[42] Toh H, Kodama J, Yanagisako M, Ohmori T (1992). Anatomical study of the accessory mental foramen and the distribution of its nerve. Okajimas Folia Anat Jpn.

[43] Please cite this paper as: Khojastepour L, Mirbeigi S, Mirhadi S, Safaee A (2015). Location of Mental Foramen in a Selected Iranian Population: A CBCT Assessment. Iran Endod J.

